# Immune checkpoint inhibitors in gynecologic oncology: Current status and perspectives

**DOI:** 10.1002/ijgo.70280

**Published:** 2025-09-04

**Authors:** Nozomu Yanaihara, Ka Yu Tse, Sung Jong Lee, Ji Geun Yoo, Sarikapan Wilailak

**Affiliations:** ^1^ Department of Obstetrics and Gynecology The Jikei University School of Medicine Tokyo Japan; ^2^ Department of Obstetrics and Gynaecology The University of Hong Kong Pokfulam Hong Kong; ^3^ Department of Obstetrics and Gynecology Seoul St. Mary‘s Hospital, The Catholic University of Korea Seoul Republic of Korea; ^4^ Department of Obstetrics and Gynecology Daejeon St. Mary‘s Hospital, The Catholic University of Korea Daejeon Republic of Korea; ^5^ Department of Obstetrics and Gynecology Faculty of Medicine Ramathibodi Hospital, Mahidol University Bangkok Thailand

**Keywords:** cervical cancer, endometrial cancer, immune checkpoint inhibitors, ovarian cancer

## Abstract

Immune checkpoint inhibitors (ICIs) have transformed cancer treatment by leveraging the immune system's capacity to fight gynecologic cancer. This review summarizes the current status and future perspectives of ICIs in the treatment of cervical, endometrial, and ovarian cancers and rare tumors. ICIs have demonstrated significant efficacy in tumors with high tumor mutational burden and immune markers such as PD‐L1 expression and microsatellite instability. In cervical cancer, the integration of ICIs has shown promise at various stages of treatment, including advanced and recurrent settings. In endometrial cancer, molecular classification has facilitated targeted immunotherapy strategies, with notable success in mismatch repair‐deficient (dMMR) tumors. However, challenges remain in the treatment of microsatellite stable endometrial and epithelial ovarian cancers due to their relatively low immunogenicity. Combination therapies, including ICIs with angiogenesis inhibitors, poly (ADP‐ribose) polymerase (PARP) inhibitors, or chemotherapy, are being actively investigated to improve response rates. Several phase II and case series showed promising response to ICIs in vulvar/vaginal cancer and gestational trophoblastic neoplasia, though the efficacy in genital tract melanoma is still unclear. Despite these advances, the management of immune‐related adverse events and the identification of reliable biomarkers for patient selection remain critical. ICIs are poised to redefine the therapeutic landscape of gynecologic oncology, offering hope for improved outcomes and personalized treatment strategies.

## 1 INTRODUCTION

**FIGURE 1:**
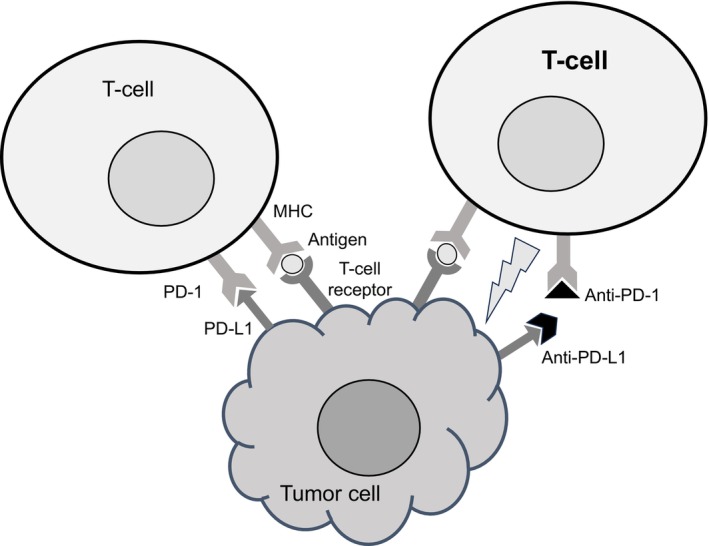
Immune checkpoint inhibitors restore the immune system's ability to recognize and attack cancer cells. PD‐1, programmed cell death protein 1; PD‐L1, programmed cell death ligand 1.

Gynecologic cancers remain one of the leading causes of death in women worldwide. Conventional multidisciplinary treatments such as surgery, radiation therapy, and chemotherapy often have limited efficacy in cases with advanced stages. The introduction of immunotherapy, particularly immune checkpoint inhibitors (ICIs), has revolutionized the treatment of gynecological malignancies, including cervical, endometrial, and ovarian cancers. ICIs work by targeting inhibitory pathways used by cancer cells to evade immune detection. Two of the most studied pathways are programmed cell death protein 1 and its ligand (PD‐1/PD‐L1) and cytotoxic T‐lymphocyte‐associated protein 4 (CTLA‐4). By inhibiting these checkpoints, ICIs restore the immune system's ability to recognize and attack cancer cells (Figure 1). Although PD‐L1 expression in gynecologic cancers is highly variable depending on tumor stage and molecular subtype, PD‐L1 is relatively overexpressed in these cancers and is associated with poor prognosis, making it a compelling therapeutic target.^1^ In addition, while other checkpoints also contribute to immune regulation, PD‐1/PD‐L1 inhibitors are often prioritized due to their established safety profile and broader applicability across tumor types. The outcomes are particularly promising in cancers with high tumor mutational burden (TMB), high microsatellite instability (MSI‐H), and neoantigen expression. TMB is the number of mutations within a tumor genome. Tumors with high TMB generate more neoantigens, which are novel peptides presented on the tumor cell surface via MHC molecules. These neoantigens are recognized as foreign by the immune system, thus stimulating the immunogenicity of the tumors and enhancing the potential efficacy of ICIs. In gynecologic cancers, high TMB is often associated with MSI‐H or mismatch repair‐deficient (dMMR) tumors, such as endometrial cancer. In addition, the tumor microenvironment significantly influences the efficacy of treatments with ICIs.^2^

Tumors characterized by high immune infiltration, abundant neoantigen expression, and an inflammatory microenvironment are referred to as hot tumors. They often express PD‐L1, making them ideal candidates for PD‐1/PD‐L1 inhibitors. Cold tumors, on the other hand, are characterized by minimal infiltration of immune cells and lack of significant inflammation or neoantigen expression, posing a challenge for the effectiveness of ICIs. Strategies to convert cold tumors into hot tumors include combination therapies with chemotherapy, radiotherapy, or other immune‐modulating agents, such as other checkpoint inhibitors, targeted therapy, and tumor vaccines.

The use of ICIs in the treatment sequence is considered highly dependent on the effective delivery of tumor neoantigens to immune cells. Survival benefits have been demonstrated in cervical cancer by studies such as KEYNOTE A‐18^3^ and BEATcc,^4^ as well as in endometrial cancer by trials like the NRG GY‐018,^5^ RUBY,^6^ AtTEnd,^7^ and DUO‐E trials.^8^ In these trials, ICIs were initiated concurrently with chemotherapy or radiation therapy and were then continued as maintenance therapy. This approach provided an opportunity for sufficient uptake of tumor antigens released during chemotherapy or radiation. In contrast, the KEYNOTE‐B21 trial,^9^ which targeted patients with 2009 FIGO Stage I–II non‐endometrioid tumors with myometrial invasion or 2009 FIGO Stage III–IVA disease, also employed a concurrent administration of ICIs with chemotherapy but failed to show a survival benefit. This lack of efficacy was hypothesized to be due to insufficient residual macroscopic disease. A similar explanation may account for the results of the CALLA study in cervical cancer.^10^ Despite being designed similarly to the KEYNOTE A‐18 trial, the CALLA trial did not demonstrate survival benefits, possibly because it included a lower‐risk population compared to the KEYNOTE A‐18.^11^

This article reviews the mechanisms, clinical trials, and future perspectives of ICIs in the treatment of gynecologic malignancies, including cervical, endometrial, and ovarian cancers, and rare tumors.

## 2 ICIs IN CERVICAL CANCER

Cervical cancer was the fifth most common female cancer globally in 2022, with 662 301 new patients and 348 874 deaths.^12^ Despite the implementation of the HPV vaccination and screening tests, the total number of cases of cervical cancer was projected to increase to 968 803 by 2040 due to population growth.^12^ For patients with locally advanced cervical cancer (LACC) (Stage IIB–IVA), the conventional treatment is concurrent chemoradiotherapy (CCRT), including concurrent cisplatin with external beam radiotherapy (EBRT), followed by brachytherapy. However, 28%–64% of cancers still recur.^13^ For patients with metastatic or recurrent cervical cancer, paclitaxel and carboplatin (TC), with or without bevacizumab, is the standard treatment.^14^ Once the disease becomes platinum‐resistant, the objective response rate (ORR) of second‐line chemotherapy, such as topotecan, gemcitabine, and etoposide, is poor and the median progression‐free survival (PFS) is typically less than 4 months.^15^, ^16^, ^17^ More than 99% of cervical cancers are related to persistent HPV infection. The integration of HPV into the host genome often leads to overexpression of the E6 and E7 oncoproteins. Although these proteins could suppress interferon activity, they induce dendritic cells to present the infected cells to cytotoxic T‐cells.^18^ PD‐L1 on tumor cells can bind to PD‐1 on T‐cells leading to immunosuppression.^19^ It has been shown that up to 70% of cervical squamous cell carcinoma (SCC) tissues have amplification of *CD274* and *PDCD1LG2* that encode PD‐L1 and PD‐L2, respectively.^20^, ^21^ In fact, over half of cervical SCC and approximately 10% of adenocarcinoma have PD‐L1 expression.^22^, ^23^, ^24^ Chronic HPV infection might also induce PD‐L1 expression.^25^, ^26^ It was shown that cervical cancer had a moderate amount of neoantigens and TMB among all cancers,^27^, ^28^ while some cervical cancers contained tumor‐infiltrating immune cells like activated memory CD4^+^ T‐cells, which could further modulate the tumor microenvironment.^29^ These make cervical cancer potentially amenable to ICIs. Selected studies are listed in Table 1 and some pivotal studies are further discussed below.

**TABLE 1:** Selected studies with results of ICIs in cervical cancer.

Trials	No.	Agents	Phase	ORR (%)	DOR (months)	mPFS (months)	PFS HR (95% CI)	mOS (months)	OS HR (95% CI)
*Recurrence after first‐line therapy*									
KEYNOTE‐028 (NCT02054806)	24	1. Pembrolizumab (10 mg/kg every 2 weeks)	Ib	17	5.4	NA			
KEYNOTE‐158 (NCT02628067)	98	1. Pembrolizumab	II	12.2	NR	NA			
EMPOWER‐Cervical 1/GOG‐3016/ENGOT‐cx9 (NCT03257267)	304 304	1. Single‐agent chemotherapy 2. Cemiplimab	III	6.3 16.4	6.9 16.4	2.8 2.9	– 0.75 (0.63–0.89)	8.5 12.0	– 0.69 (0.56–0.84)
JapicCTI‐163 212	20	1. Nivolumab	II	25	NE	5.6		N	
C‐700‐01 (NCT03104699)	161	1. Balstilimab	II	15.0	15.4				
YH‐S001‐05 (NCT03972722)	105	1. Zimberelimab	II	27.6	NR	3.7		16.8	
NRG‐GY002 (NCT02257528)	25	1. Nivolumab	II	4%	3.8				
Checkmate‐358 (NCT02488759)	15 27 43	1. Nivolumab 2. NIVO3 + IPI1^a^ 3. NIVO1 + IPI3^b^	I/II	27 26 3	NR 21.1 NR	5.5 3.6 4.7		21.9 10.3 19.9	
COMPASSION‐03 (NCT03852251)	111	1. Cadonilimab	II	32.3	NR				
SKYSCRAPER‐04 (NCT04300647)	127 45	1. Atezolizumab 2. Atezolizumab + Tiragolumab	II	15.6 19.0	NE 11.8	1.9 2.8		10.6 11.1	
C‐550‐01 (NCT03495882)	125	1. Balstilima + Alifrelimab	II	25.6	NR				
B2019‐204‐01 (NCT04341883)	27	1. Sintilimab + Nab‐paclitaxel	II	44.4	3.8	5.2		13.1	
HLX10‐011‐CC201 (NCT04150575)	21	1. Serplulimab + Nab‐paclitaxel	II	57.1	NR	5.7		15.5	
CLAP (NCT03816553)	45	1. Camrelizumab + Apatinib	II	55.6	16.6	8.9		20.3	
2018‐013‐00CH3 (NCT03903705)	27	1. Sintilimab + Fruquintinib	II	29.6	–	8.2			
ALTER‐C201 (ChiCTR1900023015)	42	1. Sintilimab + Anlotinib	II	53.8	19			17.6	
ALARICE (NCT03826589)	19	1. Avelumab + Axitinib	II	33.30	14.3	10.7		20.9	
PEVOsq (NCT04357873)	25	1. Pembrolizumab + Vorinostat	II	39.1	15.2	4.2		10.3	
InnovaTV 205/GOG‐3024/ENGOT‐cx8 (NCT03786081)	34	1. Pembrolizumab + Tisotumab vedotin	I/II	35.3	14.1	5.6		15.3	
NCT04405349	47	1. Atezolizumab + VB10.16 vaccine	II	19	–	4.1		16.9	
WBH‐7209 (NCT04096911)	13	1. Sintilimab + quadrivalent HPV vaccine at 0, 2, 6 months	II	53.8	NR	7.2		NR	
R2810‐ONC‐ISA‐1981 (NCT04646005)	113	1. Cemiplimab + ISA101b	II	16.8	5.6	3.0		13.3	
KEYNOTE‐567 (NCT03444376)	36	1. Pembrolizumab + GX‐188E	II	24 weeks: 42	4.0	4.9		10.2	
C‐145‐04 (NCT03108495)	189	Cohort 1 and 2: Lifileucel (LN‐145) Cohort 3: Pembrolizumab + LN‐145	II	Cohort 1: 44	–				
*First‐line metastatic/recurrence*									
KEYNOTE‐826 (NCT03635567)	309 308	1. TP/TC ± Bev 2. Pembrolizumab + TP/TC ± + Bev	III	51.5 66.2	10.4 18.0	8.2 10.4	– 0.61 (0.50–0.74)	16.8 26.4	– 0.63 (0.52–0.77)
BEATcc/ENGOT‐Cx10/GEICO 68‐C/JGOG1084/GOG‐3030 (NCT03556839)	204 206	1. TP/TC + Bev 2. Atezolizumab + TP/TC ± + Bev	III	72 84	8.6 13.6	10.4 13.7	– 0.62 (0.49–0.78)	22.8 32.1	– 0.68 (0.52–0.88)
JS001‐ISS‐CO214 (NCT04973904)	24	1. Toripalimab + TP/TC + Bev	II	83.3	NR	22.6		NR	
QL1604‐301 (NCT04864782)	46	1. QL1604 + TP/TC	II	58.7	9.6	8.1		NE	
CAESURA (NCT03912402)	58	1. Prolgolimab + TP/TC + Bev	II	63.8		8		NR	
BGB‐A317‐2007 (NCT05247619)	24	1. Tislelizumab + TP/TC + Bev	II	73.7	NR				
NCT03367871	17	1. Pembrolizumab + TP/TC + Bev	II	75		48.7		58.3	
AK104‐210 (NCT04868708)	16 15 13	1. AK104 10 mg/kg + TP/TC 2. AK104 15 mg/kg + TP/TC 3. AK104 10 mg/kg + TP/TC + Bev	II	68.8 73. 92.3					
Checkmate‐358 (NCT02488759)	4 18 69	1. Nivolumab 2. NIVO3 + IPI1^a^ 3. NIVO1 + IPI3^b^	I/II	25 39 41	NA 34.6 25.6	NA 17.1 7.0		NA 36.2 20.9	
COMPASSION‐13/AK104‐210‐AU (NCT04380805)	15 16 14	1. Cadonilimab 2. Cadonilimab + TP/TC 3. Cadonilimab + TP/TC + Bev	II	66.7 68.8 92.3	7.6 NR NR	11. 7.06 NR		NR NR NR	
COMPASSION‐16 (NCT04982237)	223 222	1. Placebo + TP/TC ± Bev 2. Cadonilimab + TP/TC ± Bev	III		8.2 13.2	8.1 12.7	– 0.62 (0.49–0.80)	22.8 NR	– 0.64 (0.48–0.86)
InnovaTV205/GOG‐3024/ENGOT‐cx8 (NCT03786081)	33 32	1. Tisotumab vedotin + Carboplatin 2. Tisotumab vedotin + Pembrolizumab	I/II	54.5 40.6	8.6 NR	6.9 5.3		NR NR	
*Neoadjuvant*									
NRG‐GY017 (NCT03738228)	19 17	1. Atezolizumab on days −21, 0, 21 + CCRT 2. Atezolizumab on days 0, 21, 42 + CCRT	I	pCR: 69 pCR: 40		2‐year DFS rate: 79 2‐year DFS rate: 59			
*LACC with CCRT*									
ENGOT‐cx11/GOG‐3047/KEYNOTE‐A18 (NCT042221945)	531 529	1. Placebo + CCRT 2. Pembrolizumab + CCRT	III	76 79	NR NR	2‐year PFS rate: 57 2‐year PFS rate: 68	– 0.70 (0.55–0.89)	2‐year OS rate: 81 2‐year OS rate: 87	– 0.73 (0.49–1.07)
CALLA (NCT03830866)	385 385	1. Placebo + CCRT 2. Durvalumab + CCRT	III	81 83	NE NE	2‐year PFS rate: 62 2‐year PFS rate: 66	– 0.84 (0.65–1.08)	NR NR	– 0.78 (0.55–1.10)
TRACE (ChiCTR2000032879)	22	1. Toripalimab on days 1, 22, 43 + CCRT	I/II	100	–	2‐year PFS rate: 82		2‐year OS rate: 90.9	
NICOL (NCT03298893)	16	1. Nivolumab + CCRT	I	93.8	–	2‐year PFS rate: 75			
M2019482 (NCT04368273)	30	1. Toripalimab every 2 weeks + CCRT	I	100	–				
*Maintenance after CCRT*									
GOG9929 (NCT01711515)	21	1. CCRT, then Ipilimumab	I		–	1‐year PFS rate: 81		1‐year OS rate: 90	

Abbreviations: Bev, bevacizumab; CCRT, chemoradiotherapy; DFS, disease‐free survival; DOR, duration of response; HR, hazard ratio; mOS, median overall survival; mPFS, median progression‐free survival; NA, not applicable; NE, not estimable; NR, not reached; ORR, objective response rate; pCR, pathological complete remission rate; TC, paclitaxel and carboplatin; TP, paclitaxel and cisplatin.

^a^ NIVO3 + IPI1: 3 mg/kg nivolumab every 2 weeks plus 1 mg/kg ipilimumab every 6 weeks.

^b^ NIVO1 + IPI3: 1 mg/kg nivolumab plus 3 mg/kg ipilimumab every 3 weeks for four cycles, ±240 mg nivolumab every 2 weeks (expansion group).

### 2.1 Metastatic/recurrent cervical cancer

The KEYNOTE‐028 study was one of the earliest studies that demonstrated the efficacy of ICI in metastatic and recurrent cervical cancer after first‐line treatment.^30^ Using 10 mg/kg pembrolizumab every 2 weeks for up to 24 months in PD‐L1 positive metastatic pre‐treated cervical cancer, the ORR was 17% (95% confidence interval [CI] 5–37) with a disease‐control rate (DCR) of 30%. The phase II KEYNOTE‐158 trial also showed a similar ORR (14.6%, 95% CI 7.8–24.2) and durable response with pembrolizumab monotherapy in PD‐L1 positive, previously treated advanced cervical cancer, with tolerable side effects.^31^ Because of the encouraging results of the KEYNOTE‐158 trial, the U.S. Food and Drug Administration (FDA) approved the use of pembrolizumab in metastatic/recurrent PD‐L1 positive cervical cancer after first‐line therapy in 2018. The phase III EMPOWER‐CERVICAL 1/GOG‐3016/ENGOT‐CX9 study was the first study that showed a significant overall survival (OS) benefit using anti‐PD1 (cemiplimab) in this setting.^24^

However, the survival benefits of most anti‐PD1/PD‐L1 monotherapies remained limited. Extensive efforts have been made to combine different agents with anti‐PD1/PD‐L1, including the exploration of dual blockade of immune checkpoints beyond PD‐1/PD‐L1. The CheckMate‐358 targeted both PD‐L1 and CTLA‐4, and demonstrated an ORR of 40% in patients with HPV‐positive/unknown cervical cancer using NIVO1 plus IPI3 regimen (i.e. 1 mg/kg nivolumab plus 3 mg/kg ipilimumab every 3 weeks for four cycles followed by 240 mg nivolumab every 2 weeks), in comparison to 26% with nivolumab alone.^32^, ^33^ Another phase II study targeting PD‐1 and CTLA‐4 (balstilimab and zalifrelimab) showed a promising ORR. This regimen is now being further investigated in the RaPiDS study.^34^ Further studies have been conducted to evaluate other immune checkpoints. The SKYSCRAPER‐04 study compared atezolizumab (anti‐PD‐L1) with and without tiragolumab (anti‐TIGIT [T‐cell immunoreceptor with Ig and ITIM domains]), in patients with metastatic or recurrent PD‐L1 positive cervical cancer.^35^ However, it showed an ORR increase of only 3.4% with dual blockade, while the median PFS (mPFS) and OS were similar in both groups. In addition, there are many studies attempting to combine anti‐PD1/PD‐L1 with different targeted therapy, such as tyrosine kinase inhibitors,^36^, ^37^, ^38^, ^39^, ^40^ poly (ADP‐ribose) polymerase (PARP) inhibitors (PARPi), chemotherapy,^41^, ^42^ antibody‐drug conjugate,^43^ HPV vaccine,^44^, ^45^, ^46^, ^47^ and tumor infiltrating lymphocytes.^48^ Most of these studies are at phase II and still ongoing (Table 2). More mature results and large‐scale studies are required.

**TABLE 2:** Selected ongoing studies of ICIs in cervical cancer.

Trials	Cell types	Phase	Primary endpoint	Maximum duration of ICIs	Arms	*n*	Status
*Recurrence after first‐line therapy*							
STUDY00003849 (NCT04865887)	SCC, AC, ASC	II	ORR	Not specified	1. Pembrolizumab + Lenvatinib	35	Recruiting
B2020‐229‐01 (NCT04651127)	SCC, AC, ASC	I/II	Safety, ORR	Until PD, unacceptable toxicity, patients' withdrawal, or death	1. Toripalimab + Chidamide	40	Unknown
2019‐JEK‐DIA‐001 (NCT04483544)	SCC, AC, ASC	II	ORR	Not specified	1. Pembrolizumab + Olaparib	48	Active, not recruiting
REPACC‐3 (NCT05290935)	SCC, AC, ASC	II	ORR	24 months	1. Camrelizumab + Nab‐paclitaxel	122	Unknown
RaPiDS / GOG‐3028 (NCT03894215)	SCC, AC, ASC	II	ORR	24 months	1. Balstilimab 2. Balstilimab + Zalifrelimab	12	Ongoing
*First‐line metastatic/recurrence*							
HLX10IIT05 (NCT05444374)	SCC, AC, ASC	II	ORR	2 years (35 cycles)	1. Serplulimab ++ TP + Bev	48	Not recruiting
Fermataz (NCT03912415)	SCC	III	OS	Until PD, unacceptable toxicity, patients' withdrawal, or death	1. TP/TC ± Bev 2. Prolgolimab+TP/TC ± Bev	316	Ongoing
B2021‐145‐01 (NCT04974944)	SCC PD‐L1 positive	II	PFS	Until PD, unacceptable toxicity, patients' withdrawal, or death	1. TP/TC ± Bev 2. Camrelizumab and apatinib	172	Unknown
*Neoadjuvant*							
NACI‐CERV‐003 (NCT06288373)	SCC, AC, ASC; PD‐L1 positive 1B3, 2A2, >4 cm 2B	III	ORR	Not specified	1. CCRT 2. Camrelizumab + Cisplatin + Nab‐paclitaxel, then RH and PLND	440	Recruiting
B2020‐201‐01 (NCT04799639)	SCC, AC, ASC 1B3 or 2A2	II	pCR	Not specified	1. 3 cycles of neoadjuvant sindilimab + TP, then surgery	47	Completed recruitment
MITO CERV 3 (NCT04238988)	SCC, AC, ASC 1B2‐2B	II	2‐year PFS	35 cycles for patients with risk factors	1. 3 cycles of neoadjuvant pembrolizumab + TC, then surgery	45	Ongoing
*LACC with CCRT*							
ATEZOLACC (NCT03612791)	SCC, AC, ASC IB1‐2A (PLN+), 2B‐4A (any PLN status), 4B (limited to PALN)	II	PFS	20 cycles	1. Placebo + CCRT 2. Atezolizumab + CCRT	189	Active, not recruiting
AK104‐305 (NCT05235516)	SCC, AC, ASC 3A‐4A	III	PFS	Not specified	1. Placebo + CCRT 2. Cadonilimab + CCRT	636	Active, not recruiting
NCT05084677	SCC, AC, ASC 3‐4A	II	ORR	1 year	1. Toripalimab + CCRT	96	Recruiting
IMURADIO3 (NCT05311566)	SCC, AC, ASC 1B2‐3B	II	3‐year OS rate	Until PD or intolerable side effects	1. Camrelizumab + CCRT	92	Recruiting
*Maintenance after CCRT*							
ATOMICC/GEICO 78‐C (NCT03833479)	SCC, AC, ASC 1B2, 2A2, 2B (PLN+), 3A‐4A, any stage with PALN+	II	PFS	24 months	1. CCRT 2. CCRT, then Dostarlimab	134	Active, not recruiting
eVOLVE‐Cervical/ GOG‐3092/ENGOTcx19/GEICO (NCT06079671)	SCC, AC, ASC stage 3A‐4A	III	PFS	24 months	1. Placebo 2. Volrustomig	800	Recruiting

Abbreviations: AC, adenocarcinoma; ASC, adenosquamous carcinoma; Bev, bevacizumab; CCRT, chemoradiotherapy; ICI, immune checkpoint inhibitor; N, number; ORR, objective response rate; OS, overall survival; PALN, para‐aortic lymph node; pCR, pathological complete remission rate; PD, progressive disease; PLN(D), pelvic lymph node (dissection); RH, radial hysterectomy; SCC, squamous cell carcinoma; TC, paclitaxel and carboplatin; TP, paclitaxel and cisplatin.

### 2.2 Advanced/recurrent cervical cancer at first‐line setting

The promising results of ICIs in the recurrent setting led to an interest in extending these agents in the frontline setting. The KEYNOTE‐826^49^, ^50^ and BEATcc^4^ integrated ICIs in cisplatin (or carboplatin), paclitaxel, with or without bevacizumab, for patients with persistent, metastatic, or recurrent cervical cancer. Both studies showed a significant improvement in mPFS of 2–3 months and median OS of 9 months or longer. Recently, the COMPASSION 16 study showed that the addition of cadonilimab, a PD‐1/CTLA‐4 bispecific antibody, with traditional platinum‐based chemotherapy could lead to significantly longer mPFS (12.7 vs. 8.1 months; hazard ratio [HR] 0.62, 95% CI 0.49–0.80) and median OS (not reached vs. 22.8 months; HR 0.64, 95% CI 0.48–0.86) compared to chemotherapy alone. The treatment‐related adverse events (TRAEs) were similar in both groups.^51^

### 2.3 Locally advanced cervical cancer in a frontline setting

CCRT can induce DNA damage and cell death, trigger cytokine release, modulate the T‐cell repertoire, and potentially synergize with ICIs.^52^, ^53^ The recent KEYNOTE‐A18 study randomized 1060 patients with LACC, and demonstrated that pembrolizumab and CCRT improved the 24‐month PFS rate from 57% to 68% (HR 0.70, 95% CI 0.55–0.89) compared to CCRT alone.^3^ A similar study, the CALLA study that evaluated 770 patients, showed no significant different in the 12‐month PFS rates between durvalumab and placebo with CCRT in LACC (HR 0.84, 95% CI 0.65–1.08).^10^ However, in their subgroup analysis of patients with PD‐L1 tumor area positivity score of 20% or above, the mPFS was significantly longer in those patients using durvalumab compared to the placebo arm (HR 0.62, 95% CI 0.42–0.91). It was difficult to compare the discrepant results between the KEYNOTE‐A18 and CALLA studies due to the difference in study primary endpoints, definition of lymph node metastasis on radiological examination, heterogeneous population in Stage III–IV, proportions of patients with para‐aortic lymph node metastasis, and the target of the study ICIs, where pembrolizumab targets PD‐1 and durvalumab targets PD‐L1.^54^ It is noteworthy that the KEYNOTE‐A18 allowed cisplatin for the CCRT, while approximately 6% of patients received carboplatin instead of cisplatin in the CALLA study. In addition, the proportion of Asians was different in both studies (i.e. 29% in the KEYNOTE‐A18 study and 39% in the CALLA study).

Currently, there are also studies that evaluate the efficacy of neoadjuvant immunotherapy with or without chemotherapy before either surgery or CCRT. For example, the phase II NACI‐CERV‐001 showed an ORR of 98% when using cisplatin, nab‐paclitaxel, and camrelizumab before radical surgery or CCRT in patients with Stage I B3, IIA2, larger than 4 cm IIB/IIIC1r, PD‐L1 positive cervical cancer.^55^ The phase III NACI‐CERV‐003 (NCT06288373) is now ongoing and recruitment is expected to be completed in 2031.

### 2.4 Challenges and future perspectives

PD‐L1 expression is a prognostic marker for cervical cancer.^22^ It is also a major predictive biomarker used for anti‐PD1/PD‐L1. Different ICIs have different companion PD‐L1 assays and scoring systems (Table 3). Among the antibodies, 22C3 appeared to be the most sensitive.^56^, ^57^ The combined positivity score (CPS) is the most common scoring method in cervical cancer. The score is determined by the ratio of PD‐L1 positive cells (including both tumor and immune cells) to the total number of viable tumor cells, multiplied by 100, and assessed using the cell count method. Most studies used a CPS of 1 or above as the cutoff in cervical cancer. However, this scoring system could be subjective. The overtly high proportion of cervical cancer with a CPS of 1 or above also makes it difficult to select appropriate patients for ICIs. Therefore, other studies, like the CheckMate 358, KEYNOTE‐826, and NACI‐CERV‐001 studies, also evaluated the efficacy of ICIs using a cutoff value of 10% or above. Some studies utilized tumor area positivity, which is determined by the area occupied by PD‐L1 positive tumor and immune cells in a given tumor area and is scored by visual‐based estimation. It might have a higher concordance rate among pathologists compared to the CPS score,^58^ but there is no consensus on the best cutoff value in cervical cancer.^10^, ^35^ In addition, the timing of cervical tissue biopsy and use of previous treatment might also affect the PD‐L1 expression.^59^

**TABLE 3:** Companion PD‐L1 assays for ICIs in selected studies.

Studies	ClinicalTrials.gov identifier	Immunotherapy	PD‐L1 assay	Scoring method	Cutoffs studied
BEATcc	NCT03556839	Atezolizumab	? SP263 (Ventana Medical System)	? TAP	Not specified
Skyscraper‐04	NCT04300647	Atezolizumab	SP263 (Ventana Medical System)	TAP	5%–9%, ≥10
ALARICE	NCT03826589	Avelumab	22C3 pharmDx assay (Agilent Technologies)	CPS	<1, ≥1–10, ≥10
C‐550‐01	NCT03495882	Balstilimab	22C3 pharmDx assay (Agilent Technologies)	CPS	<1, ≥1
C‐700‐01	NCT03104699	Balstilimab	22C3 pharmDx assay (Agilent Technologies)	CPS	<1, ≥1
COMPASSION‐13	NCT03852251	Cadonilimab	22C3 pharmDx assay (Agilent Technologies)	CPS	<1, ≥1–10, ≥10
COMPASSION‐16	NCT04982237	Cadonilimab	22C3 pharmDx assay (Agilent Technologies)	CPS	<1, ≥1–10, ≥10
CLAP	NCT03816553	Camrelizumab	22C3 pharmDx assay (Agilent Technologies)	CPS	<1, ≥1–10, ≥10
NACI‐CERV‐001	NCT04516616	Camrelizumab	22C3 pharmDx assay (Agilent Technologies)	CPS	≥10, 20, 30, 40, and 50
EMPOWER‐Cervical‐1	NCT03257267	Cemiplimab	SP263 (Ventana Medical System)	Tumor cell expression score^a^	<1, ≥1
R2810‐ONC‐ISA‐1981	NCT04646005	Cemiplimab	SP263 (Ventana Medical System)		<1, ≥1
CALLA	NCT03830866	Durvalumab	SP263 (Ventana Medical System)	TAP	<1, ≥1, ≥5, ≥20
CheckMate‐358	NCT02488759	Nivolumab	28–8 pharmDx (Dako–Agilent Technologies)	Tumor cell expression score^a^	<1, ≥1
CheckMate‐358	NCT02488759	Nivolumab	28–8 pharmDx (Dako–Agilent Technologies)	CPS	<1, ≥1, <10, ≥10
JapicCTI‐163 212	JapicCTI‐163 212	Nivolumab	28–8 pharmDx (Dako–Agilent Technologies)	CPS	<1, ≥1
NRG‐GY002	NCT02257528	Nivolumab	E1L3N (Cell Signaling Technologies)	CPS	<1, ≥1
KEYNOTE‐028	NCT02054806	Pembrolizumab	22C3 (QualTek Molecular Laboratories)^b^	Modified proportion score	<1, ≥1
KEYNOTE‐158	NCT02628067	Pembrolizumab	22C3 pharmDx assay (Agilent Technologies)	CPS	<1, ≥1
KEYNOTE −826	NCT03635567	Pembrolizumab	22C3 pharmDx assay (Agilent Technologies)	CPS	<1, 1–<10, ≥10
KEYNOTE‐A18	NCT04221945	Pembrolizumab	22C3 pharmDx assay (Agilent Technologies)	CPS	<1, ≥1
CAESURA	NCT03912402	Prolgolimab	22C3 pharmDx assay (Agilent Technologies)	CPS	<1, ≥1
QL1604‐301	NCT04864782	QL1604	SP263 (Ventana Medical System)	CPS	<1, 1–49, ≥50
HLX10‐011‐CC201	NCT04150575	Serplulimab	Not specified	CPS	1–<20, ≥20%
B2019‐204‐01	NCT04341883	Sintilimab	22C3 pharmDx assay (Agilent Technologies)	CPS	<1, ≥1
WBH‐7209	NCT04096911	Sintilimab	Not specified	CPS	≥1, <10, ≥10
TRACE	ChiCTR2000032879	Toripalimab	22C3 pharmDx assay (Agilent Technologies)	CPS	< 1, 1–4, ≥ 5
YH‐S001‐05	NCT03972722	Zimberelimab	22C3 pharmDx assay (Agilent Technologies) and WD160 (WuXi Diagnostics)	CPS	<1, ≥1

Abbreviations: CPS, combined positivity score; TAP, tumor area positivity.

^a^ The percentage of tumor cells expressing PD‐L1.

^b^ Membranous staining on ≥1% modified proportion score or interface pattern.

Some studies evaluated other biomarkers like histology. For example, the BEATcc study showed the mPFS was improved in patients with adenocarcinoma/LACC using atezolizumab with CCRT compared to CCRT (HR 0.59, 95% CI 0.45–0.76) but not SCC (HR 0.75, 95% CI 0.47–1.19).^4^ In contrast, the EMPOWER study showed that there was survival benefit in both SCC (HR 0.73, 95% CI 0.58–0.91) and adenocarcinoma/adenosquamous carcinoma comparing cemiplimab with chemotherapy in recurrent cervical cancer (HR 0.56, 95% CI 0.36–0.85).^24^ However, these results should be interpreted with caution as the studies were not powered to distinguish the difference based on these biomarkers.

Future research should focus on identifying more reliable predictive biomarkers to determine the optimal timing of ICIs with respect to conventional treatment in the frontline setting. It should also evaluate the efficacy and safety of combined treatment with ICIs, elucidate the mechanism of resistance to ICIs, and investigate optimal treatments after progression following ICIs, including the possibility for re‐challenge treatment using ICIs.

## 3 ICIs IN ENDOMETRIAL CANCER

Endometrial cancer has been classified into types I and II based on its dependency on the estrogen hormone.^60^ For high‐risk cases, adjuvant therapy, such as radiotherapy or chemotherapy, has been implemented. The GOG 258 and PORTEC‐3 studies investigated the combination of chemotherapy and radiotherapy as a treatment strategy.^61^, ^62^ Recently, a new era has emerged in which endometrial cancer is recognized as a molecularly driven malignancy. High somatic mutation rates have been observed in uterine cancer, lung cancer, and melanoma, categorizing these as immunogenic tumors, which subsequently became targets for immunotherapy.^27^ The Cancer Genome Atlas classified endometrial cancer into four subtypes using whole‐genome sequencing and compared prognoses based on mutation types.^63^ The Proactive Molecular Risk for Endometrial Cancer has improved molecular classification to make it more applicable in clinical practice: *POLE* mutated (POLEmut); dMMR; no specific molecular profile (NSMP); and p53abn.^64^ The new FIGO 2023 staging for endometrial cancer incorporates molecular classification, highlighting the role of downstaging for POLEmut and upstaging for p53abn in Stages I and II.^65^ Among them, dMMR MSI‐H subtypes are characterized by high TMB, increased infiltration of cytotoxic T‐cells, peri‐tumoral reaction, and PD‐1/PD‐L1 expression, which suggests a favorable response to ICIs.^66^, ^67^, ^68^ On the other hand, microsatellite stable (MSS) tumors show relatively low efficacy with ICIs,^69^ but have demonstrated improved anti‐cancer efficacy and the ability to overcome resistance by modifying the tumor microenvironment.^70^, ^71^, ^72^

### 3.1 ICIs in the first‐line treatment of endometrial cancer

Chemotherapy not only exerts direct cytotoxic effects on tumor cells but also activates the immune system within the tumor microenvironment through several mechanisms, including reducing regulatory T‐cells and immunosuppressive myeloid‐derived stem cells, inducing tumor‐infiltrating lymphocytes, and upregulating PD‐L1 expression on tumor cells.^73^ PD‐1/PD‐L1 inhibitors (dostarlimab, pembrolizumab, durvalumab, and atezolizumab) have demonstrated their efficacy when combined with TC as the first‐line treatment for endometrial cancer through randomized phase III clinical trials: RUBY; NRG‐GY018; DUO‐E; and AtTEnd.^5^, ^6^, ^7^, ^8^ Their greatest benefit was demonstrated with a HR in the range of 0.3–0.45 in the dMMR group compared to chemotherapy alone. These study results have contributed to the selection of immunotherapy (PD‐1 or PD‐L1 antibodies) and chemotherapy as first‐line treatments for endometrial cancer (Table 4).

**TABLE 4:** Clinical trials of ICIs in first‐line treatment setting of endometrial cancer.

Trials	Eligibility (*n*)	Arms	Phase	mPFS (months)	PFS HR (95%CI) or ORR (%)
MITO END‐3 (NCT03503786)	Advanced or recurrent EC (125)	1. TC 2. TC + Avelumab + Maint	II	9.9 9.6	– 0.78 (0.65–0.93)
NRG‐GY‐018 (NCT03914612)	Advanced or recurrent EC (816)	1. TC + Placebo + Maint 2. TC + Pembrolizumab + Maint	III	dMMR: 7.6, pMMR: 8.7 dMMR: NR, pMMR: 13.1	– dMMR: 0.30 (0.19–0.48), pMMR: 0.54 (0.41–0.71)
RUBY (Part 1) (NCT03981796)	Advanced or recurrent EC (494)	1. TC + Placebo + Maint 2. TC + Dostarlimab + Maint	III	dMMR: 7.7, pMMR: 7.9 dMMR: NR, pMMR: 9.9	– dMMR: 0.28 (0.16–0.50), pMMR: 0.76 (0.59–0.98)
AtTEnd (NCT03603184)	Advanced or recurrent EC (551)	1. TC + Placebo + Maint 2. TC + Atezolizumab + Maint	III	dMMR: 6.9, pMMR: 9.2 dMMR: NR, pMMR: 9.5	– dMMR: 0.36 (0.23–0.57), pMMR: 0.92 (0.73–1.16)
DUO‐E (NCT04269200)	Advanced or recurrent EC (718)	1. TC + Placebo + Placebo + Maint (Placebo + Placebo) 2. TC + Durvalumab + Placebo + Maint (Dostarlimab + Placebo) 3. TC + Durvalumab + Olaparib + Maint (Dostarlimab + Olaparib)	III	9.6 10.2 15.1	– 0.71 (0.57–0.89) 0.55 (0.43–0.69)
RUBY (Part 2) (NCT03981796)	Advanced or recurrent EC (291)	1. TC + Placebo + Placebo + Maint (Placebo + Placebo) 2. TC + Dostarlimab + Niraparib + Maint (Dostarlimab + Niraparib)	III	8.3 14.5	– 0.60 (0.43–0.82)
KEYNOTE‐B21 (NCT04634877)	High‐risk EC without evidence of disease (1095)	1. TC + Placebo + Maint ± Radiotherapy 2. TC + pembrolizumab + Maint +/–Radiotherapy	III	dMMR: NR, pMMR: NR dMMR: NR, pMMR: NR	– dMMR: 0.31 (0.14–0.69), pMMR: 1.20 (0.91–1.57)
LEAP‐001 (NCT03884101)	Advanced or recurrent EC (842)	1. TC 2. Lenvatinib + Pembrolizumab + Maint	III	10.2 12.5	– 0.91 (0.76–1.09)
KEYNOTE‐C93 (NCT05173987)	Advanced or recurrent EC	1. TC ± Trastuzumab (for HER2+ serous EC) 2. Pembrolizumab + Maint	III	Ongoing	Ongoing
DOMENICA (NCT05201547)	Advanced or recurrent EC	1. TC 2. Dostarlimab + Maint	III	Ongoing	Ongoing
NRG‐GY‐020 (NCT04214067)	Stage I/II MSI‐H/dMMR EC with high‐intermediate risk	1. EBRT + brachytherapy 2. Vaginal brachytherapy/pelvic radiation + Pembrolizumab	III	Ongoing	Ongoing

Abbreviations: dMMR, mismatch repair deficiency; EC, endometrial cancer; HER2, human epidermal growth factor receptor 2; HR, hazard ratio; ICI, immune checkpoint inhibitor; Maint, maintenance; mPFS, median progression‐free survival; MSI‐H, microsatellite instability‐high; NR, not reached; ORR, objective response rate; pMMR, mismatch repair proficiency; TC, paclitaxel and carboplatin.

The four studies share similarities but also exhibit notable differences. When comparing NRG‐GY018 and RUBY, key distinctions include the enrollment time point after chemotherapy completion (12 months vs. 6 months) and the duration of immunotherapy treatment (2 years vs. 3 years). In the PFS analysis, NRG‐GY018 performed a separate evaluation of dMMR and mismatch repair‐proficient (pMMR) populations, whereas RUBY focused on the overall population with subgroup analyses for dMMR and pMMR. Regarding racial composition, both the AtTEnd and DUO‐E trials included more than 20% Asian participants, highlighting the importance of considering potential ethnic variations in treatment outcomes. The RUBY, DUO‐E, and AtTEnd trials included patients with carcinosarcoma. TRAEs were more frequent in patients who were treated with ICIs, but most immune‐related adverse events (irAEs) were grade 1 or 2 and manageable.^74^ With this greater magnitude of survival benefit, ICIs have become established as the first‐line treatment for dMMR/MSI‐H advanced or metastatic endometrial cancer.

For patients with pMMR/MSS endometrial cancer, the LEAP‐001 study, a randomized phase III trial, compared a chemotherapy‐free combination of pembrolizumab plus lenvatinib with TC as the first‐line therapy. This combination had previously demonstrated superior efficacy over conventional chemotherapy in the second‐line treatment of the KEYNOTE‐775 study. However, pembrolizumab plus lenvatinib did not show significant benefits compared with chemotherapy in PFS or OS in pMMR populations.^75^ Another strategy involves adding PARPi to the combination of chemotherapy and ICIs, as demonstrated in the DUO‐E and RUBY (Part 2) trials. In the pMMR subgroup of the DUO‐E trial, the PFS HR for durvalumab plus olaparib arm compared to the control arm and durvalumab arm was 0.57 (95% CI 12.4–18.0) and 0.76 (95% CI 0.59–0.99), respectively.^8^ In the RUBY (Part 2) trial, the PFS HR for dostarlimab plus niraparib arm in the pMMR subgroup compared to the control arm was 0.63 (95% CI 0.44–0.91; *P* = 0.006), showing a significant benefit.

The aforementioned studies primarily focused on a non‐curative setting, with most patients having measurable disease. In contrast, the phase III randomized KEYNOTE‐B21 trial evaluated pembrolizumab plus chemotherapy in patients with newly diagnosed high‐risk endometrial cancer, such as Stage I–II endometrioid type with p53 abnormality or Stage III–IVa without macroscopic residual burden.^9^ In the dMMR subgroup, the HR for disease‐free survival favored the pembrolizumab group (HR 0.31, 95% CI 0.14–0.69), showing results comparable to those of the NRG‐GY018 study. However, no clinical benefit was observed in the pMMR subgroup (HR 1.20, 95% CI 0.91–1.57), a finding that diverges from the clinically meaningful improvement reported in the NRG‐GY018 study. Although the reasons for this discrepancy remain unclear, it suggests that factors such as tumor burden, proportion of advanced disease stage, somatic gene alterations, and heterogeneity within pMMR groups may influence the response to immunotherapy.

### 3.2 ICI in the second‐line treatment of endometrial cancer

Pembrolizumab received tissue‐agnostic approval for patients with advanced dMMR/MSI‐H tumors, including endometrial cancer, based solely on biomarker status (Table 5). The ORR was 48% (95% CI 37–60), with the duration of response lasting over 1 year in 88% and over 3 years in 68%.^76^ Another PD‐1 inhibitor, dostarlimab, also demonstrated its efficacy and safety in the phase I GARNET trial. It showed an ORR of 45.5% (95% CI 37–54), with a duration of response lasting over 1 year in 93.3% and over 2 years in 83.7%.^77^

**TABLE 5:** Clinical trials of ICIs in second‐line treatment setting of endometrial cancer.

Trials	Eligibility (*n*)	Arms	Phase	mPFS (months)	PFS HR (95% CI) or ORR (%)
KEYNOTE‐028 (NCT02054806)	PD‐1‐positive EC (24)	Pembrolizumab	Ib	1.8	ORR 13
KEYNOTE‐158 (NCT02628067)	MSI‐H/dMMR EC (90)	Pembrolizumab	II	13.1	ORR 48
Konstantinopoulos et al. (NCT02912572)	MSI‐H/dMMR EC (15) pMMR/MSS EC (16)	Avelumab	II	4.4 1.9	ORR 26.7 ORR 6.3
GARNET (NCT02715284)	MSI‐H/dMMR EC (143) pMMR/MSS EC (156)	Dostarlimab	I/II	6.0 2.7	ORR 45.5 ORR 15.4
PHAEDRA (NCT03015129)	MSI‐H/dMMR EC: 0–3 prior lines of chemotherapy (35) pMMR/MSS EC: 1–3 prior lines of chemotherapy (36)	Durvalumab	II	8.3 1.8	ORR 47 ORR 3
PHAEDRA (NCT03015129)	Previously treated recurrent or persistent EC (77) dMMR (9), pMMR (63)	1. Durvalumab 2. Durvalumab + Tremelimumab	II	1.7 1.8	ORR 10.8 ORR 5.3
KEYNOTE‐775 (NCT03517449)	Previously treated recurrent or persistent EC (827) dMMR (130), pMMR (697)	1. Physician's choice CX 2. Lenvatinib + Pembrolizumab	III	3.8 7.3	– 0.56 (0.48–0.66)
Fuh et al. (NCT03526432)	Previously treated recurrent or persistent EC (57) dMMR (13%), pMMR (87%)	Atezolizumab + Bevacizumab	II	7.9	ORR 30

Abbreviations: CX, chemotherapy; dMMR, mismatch repair deficiency; EC, endometrial cancer; HR, hazard ratio; ICI, immune checkpoint inhibitor; mPFS, median progression‐free survival; MSI‐H, microsatellite instability‐high; MSS, microsatellite stable; ORR, objective response rate; PD‐1, programmed cell death protein 1; pMMR, mismatch repair proficiency.

The phase III STUDY 309/KEYNOTE‐775 randomized pembrolizumab and lenvatinib versus the physician's choice of chemotherapy to patients with measurable metastatic or recurrent endometrial cancers, as determined by the Response Evaluation Criteria in Solid Tumors, despite prior chemotherapy. Lenvatinib, an oral tyrosine kinase inhibitor, targets vascular endothelial growth factor receptors and fibroblast growth factor receptors. Pembrolizumab plus lenvatinib showed a significantly longer OS (HR 0.70, 95% CI 0.58–0.83) and PFS (HR 0.60, 95% CI 0.50–0.72) compared to chemotherapy.^78^ Adverse events of grade 3 or higher occurred in 88.9% of patients receiving pembrolizumab plus lenvatinib. The combination of these two agents has sparked interest in managing overlapping toxicities. Since lenvatinib is taken orally daily and pembrolizumab is administered intravenously every 3 weeks, it is recommended to monitor the timing of onset and the characteristics of symptoms related to these adverse effects. The starting dose of lenvatinib is 20 mg, and reducing the dose from the outset may impact its efficacy. If adverse effects become difficult to manage, it is recommended to adjust the lenvatinib dose first, as it is taken daily, before making any adjustments to pembrolizumab.^79^

### 3.3 Future perspectives

Molecular characterization in endometrial cancer has led to paradigm shifts, establishing immunotherapy as one of the primary treatment options.^80^ The MMRd‐GREEN trial of the RAINBO program compares adjuvant radiotherapy plus durvalumab to radiotherapy alone in patients with Stage II or III dMMR endometrial cancer.^81^ The KEYNOTE‐C93 and DOMENICA studies are ongoing to determine the possibility of substituting chemotherapy with pembrolizumab and dostarlimab monotherapy, respectively, as first‐line treatments for dMMR expressing advanced or recurrent endometrial cancer.^82^, ^83^ In addition to immunotherapy, ongoing studies on oral selinexor, as well as antibody drug‐conjugates targeting HER2, folate receptor α, and TROP2, are expected to bring significant advancements in the treatment of endometrial cancer.

## 4 ICIs IN OVARIAN CANCER

Despite advances in surgical techniques and the development of multidimensional drug therapies using chemotherapy and molecularly targeted agents, epithelial ovarian cancer (EOC) still has the lowest survival rate of all gynecologic malignancies, suggesting the need for innovative approaches to this type of cancer. The tumor microenvironment for EOC often promotes immune suppression, making it a key target for ICIs. However, the efficacy of ICIs in EOC is less clear compared to cervical and endometrial cancers.^84^

### 4.1 Single‐agent or double‐agent ICIs in ovarian cancer

Several clinical trials have investigated the use of ICIs, including PD‐1 inhibitors (pembrolizumab and nivolumab) and PD‐L1 inhibitors (avelumab and atezolizumab), in the treatment of EOC. Early phase trials have shown some promising results, but overall response rates have been lower than expected. Table 6 summarizes the clinical trials that evaluated the use of ICIs in the treatment of EOC, either as monotherapy or in combination with two types of ICI.

**TABLE 6:** Clinical trials of single‐agent or double‐agents ICIs in ovarian cancer.

Trials	Eligibility (*n*)	Agents	Phase	ORR (%)	mPFS (months)
KEYNOTE‐100 (NCT02674061)	PSROC+PRROC Cohort A: 1–3 prior lines and TFI 3–12 months (285) Cohort B: 4–6 prior lines and TFI >3 months (91)	Pembrolizumab (anti‐PD‐1)	II	Cohort A: 8.1 Cohort B: 9.9	A: 2.1 B: 2.1
KEYNOTE‐28 (NCT02054806)	PSROC+PRROC (26) PD‐L1 ≥ 1% in tumor and immune cells	Pembrolizumab (anti‐PD‐1)	Ib	11.5	1.9
Hamanishi et al. (UMIN000005714)	PRROC (20)	Nivolumab (anti‐PD‐1)	II	15.0	3.5
JAVELIN Solid Tumor (NCT01772004)	PSROC+PRROC (125)	Avelumab (anti‐PD‐L1)	Ib	All: 9.6 PSOC: 13.6 PROC: 5.3	2.6
NRG‐GY‐003 (NCT02498600)	PSROC+PRROC (100) PFI <12 months	1. Nivolumab 2. Nivolumab + Ipilimumab	III	12.2 31.4	2.0 3.9

Abbreviations: mPFS, median progression‐free survival; ORR, objective response rate; PFI, platinum‐free interval; PRROC, platinum‐resistant recurrent ovarian cancer; PSROC, platinum‐sensitive recurrent ovarian cancer; TFI, treatment‐free interval.

Pembrolizumab has been evaluated in platinum‐sensitive (PSROC) and platinum‐resistant (PRROC) recurrent ovarian cancer in the KEYNOTE‐100 and KEYNOTE‐28 trials.^85^, ^86^ The phase II KEYNOTE‐100 trial showed limited activity of pembrolizumab monotherapy with an ORR of 7.4% in patients with a treatment‐free interval (TFI) of 3–12 months and 9.9% in patients with a TFI longer than 3 months. The KEYNOTE‐28 trial, which used molecular biomarkers such as PD‐L1 of 1% or more in tumor and immune cells, also showed disappointing results with an ORR of 11.5% in heavily pretreated relapsed EOC patients. The study by Hamanishi et al.^87^ tested another PD‐1 inhibitor, nivolumab, as monotherapy in patients with PRROC and showed relatively favorable results with an ORR of 15.0% and an mPFS of 3.5 months. However, in the subsequent phase III NINJA trial, patients with PRROC who received nivolumab had worse PFS compared to those treated with gemcitabine or pegylated liposomal doxorubicin (2.0 months vs. 3.8 months; HR 1.50, 95% CI 11.20–1.90).^88^ In addition, the NRG‐GY‐003 trial evaluated nivolumab alone and in combination with the anti‐CTLA4 antibody, ipilimumab, in patients with PSROC and PRROC with a platinum‐free interval of less than 12 months.^89^ The ORR was 12.2% with nivolumab alone and increased to 31.4% in combination with ipilimumab. Survival was different between the two arms, with an mPFS of 3.9 months for the combination versus 2 months for nivolumab alone (HR 0.53, 95% CI 0.34–0.82). Clinical trials that tested anti‐PD‐L1 antibodies as monotherapy, including the JAVELIN Solid Tumors trial that evaluated avelumab in PSROC and PRROC and found a total ORR of 9.6%, similarly showed only inadequate efficacy in patients with recurrent ovarian cancer.^90^ Overall, these results may highlight the need for better patient selection and combination strategies to improve the efficacy of ICIs in EOC.

### 4.2 Clinical trials of ICIs in first‐line treatment setting of ovarian cancer

To improve the efficacy of ICIs in EOC, combination therapies are being actively investigated (Table 7). Promising approaches include PARPi, which target DNA repair mechanisms, and the anti‐angiogenic agent bevacizumab. Preclinical studies have suggested that combining PARPi with ICIs may enhance the immune response by increasing TMB and neoantigen production, making the tumor more susceptible to immune attack. In addition, the combination of bevacizumab with ICIs has been shown to increase immune cell infiltration into tumors, potentially overcoming immune suppression.

**TABLE 7:** Clinical trials of ICIs in first‐line treatment setting of ovarian cancer.

Trials	Eligibility (*n*)	Arms	Phase	mPFS (months)	PFS HR (95% CI)
DUO‐O/ENGOT ov‐46 (NCT03737643)	III–IV (1130) Non‐t*BRCA*m	1. TC + Bev + Maint (Bev + Placebo + Placebo) 2. TC + Bev + Durvalumab + Maint (Bev + Durvalumab + Placebo) 3. TC + Bev + Durvalumab + Maint (Bev + Durvalumab + Olaparib)	III	19.3 20.6 25.1	– 0.87 (0.74–1.03) 0.61 (0.51–0.73)
JAVELIN Ovarian 100 (NCT02718417)	III–IV (998)	1. TC 2. TC + Maint (Avelumab) 3. TC + Avelumab + Maint (Avelumab)	III	NE 16.8 18.1	– 1·43 (1·05–1·95) 1·14 (0·83–1·56)
IMagyn050/GOG‐3015/ENGOT‐ov39 (NCT03038100)	III–IV (1301)	1. TC + Bev + Maint (Bev + Placebo) 2. TC + Bev + Atezolizumab + Maint (Bev + Atezolizumab)	III	18.4 19.5	– 0.92 (0.79–1.07)
ATHENA‐COMBO/GOG‐3020/ENGOT ov‐45 (NCT03522246)	III–IV (863)	1. Maint (Placebo + Rucaparib) 2. Maint (Nivolumab + Rucaparib)	III	20.2 15.0	– 1.29 (1.08–1.53)
FIRST/ENGOT ov‐44 (NCT03602859)	III–IV	1. TC + Placebo + Maint (Placebo + Niraparib) 2. TC + Dostralimab + Maint (Dostralimab + Niraparib)	III	Ongoing	Ongoing
ENGOT ov‐43/GOG‐3036/KEYLYNK‐001 (NCT03740165)	III–IV Non‐g*BRCA*m Non‐t*BRCA*m	1. TC + Placebo + Maint (Placebo + Placebo) 2. TC + Pembrolizumab + Maint (Pembrolizumab + Placebo) 3. TC + Pembrolizumab + Maint (Pembrolizumab + Olaparib)	III	Ongoing	Ongoing

Abbreviations: Bev, bevacizumab; g*BRCA*m, germline *BRCA* mutation; HR, hazard ratio; ICI, immune checkpoint inhibitor; Maint, maintenance; mPFS, median progression‐free survival; NE, not estimable; t*BRCA*m, tumor *BRCA* mutation; TC, paclitaxel and carboplatin.

The DUO‐O trial enrolled patients with Stage III–IV ovarian cancer who did not have a *BRCA* mutation (*n* = 1130). Three first‐line treatment arms were evaluated and the experimental arm of TC chemotherapy plus bevacizumab with durvalumab followed by maintenance with the triplet regimen (bevacizumab plus durvalumab plus olaparib) showed significantly improved PFS compared to the control arm of bevacizumab alone in the intention‐to‐treat (ITT) population (25.1 months vs. 19.3 months; HR 0.61, 95% CI 0.51–0.73) as well as in the homologous recombination deficient (HRD) subgroup (45.1 months vs. 23.3 months; HR 0.46, 95% CI 0.33–0.65).^91^ Although the ideal control arm in this clinical setting would be the PAOLA‐1 regimen (TC plus bevacizumab followed by maintenance with bevacizumab plus olaparib), these results suggest that combining ICIs with two or more molecularly targeted agents in a maintenance setting may provide enhanced therapeutic benefits.

Other phase III clinical trials in the first‐line setting include the JAVELIN Ovarian 100 trial and the IMagyn050/GOG 3015/ENGOT‐OV39 trial.^92^, ^93^ The JAVELIN Ovarian 100 trial, which evaluated the addition of avelumab to TC chemotherapy in newly diagnosed Stage III–IV ovarian cancer (*n* = 998), did not meet its primary endpoints. The mPFS was 16.8 months in the avelumab maintenance arm, 18.1 months in the avelumab combination arm, and NE in the control arm. The stratified HR for PFS was 1.43 (95% CI 1.05–1.95) and 1.14 (95% CI 0.83–1.56) in the avelumab maintenance and avelumab combination arms, respectively, and was not significantly different from the control arm. Similarly, in the IMagnyn050/GOG 3015/ENGOT‐OV39 trial (*n* = 1301), there was no significant difference in PFS in the ITT population between the addition of ICI to TC chemotherapy plus bevacizumab and TC chemotherapy plus bevacizumab. The mPFS was 19.5 months in the atezolizumab arm and 18.4 months in the control arm (HR 0.92, 95% CI 0.79–1.07). In addition, the efficacy of rucaparib combined with nivolumab as maintenance therapy in patients with newly diagnosed advanced ovarian cancer (*n* = 863) was evaluated in the ATHENA‐COMBO/GOG‐3020/ENGOT ov‐45 trial.^94^ However, rucaparib in combination with nivolumab was associated with increased toxicity and did not extend the PFS benefit of rucaparib monotherapy as the first‐line maintenance therapy. Ongoing trials (FIRST/ENGOT OV‐44 and ENGOT OV‐43/GOG‐3036/KEYLYNK‐001) are evaluating different combinations of TC chemotherapy, dostarlimab, pembrolizumab, and PARPi in advanced EOC.^95^

### 4.3 Clinical trials of ICIs in recurrent setting of ovarian cancer

The ANITA/ENGOT‐ov41/GEICO 69‐O^96^ and ATALANTE/ENGOT‐ov29^97^ trials are both phase III trials evaluating standard platinum‐based chemotherapy plus atezolizumab in PSROC (Table 8). In the ANITA/ENGOT‐ov41/GEICO 69‐O study, the efficacy of adding atezolizumab to platinum‐based chemotherapy and subsequent niraparib maintenance was evaluated in 417 patients with PSROC. However, this trial did not show a significant improvement in PFS with the addition of atezolizumab (11.2 months vs. 10.1 months; HR 0.89, 95% CI 0.71–1.10). Subgroup analyses, including those in PD‐L1 positive patients, showed no additional benefit. The ATALANTE/ENGOT‐ov29 trial evaluated whether the addition of atezolizumab to standard platinum‐based chemotherapy plus bevacizumab could improve PFS in a similar patient population (*n* = 614). Like the ANITA/ENGOT‐ov41/GEICO 69‐O trial, it did not meet its co‐primary endpoints, as the addition of atezolizumab did not significantly improve PFS either the overall (13.5 months vs. 11.3 months; HR 0.83, 95% CI 0.69–0.99) or PD‐L1 positive (15.2 months vs. 13.1 months; HR 0.86, 95% CI 0.63–1.16) population.

**TABLE 8:** Clinical trials of ICIs in recurrent setting of ovarian cancer.

Trials	Eligibility (*n*)	Arms	Phase	mPFS (months)	PFS HR (95% CI) or ORR (%)
ANITA/ENGOT ov‐41/GEICO 69‐O (NCT03598270)	PSROC (417)	1. Platinum‐based CX + Placebo + Maint (Placebo + Niraparib) 2. Platinum‐based CX + Atezolizumab + Maint (Atezolizumab + Niraparib)	III	10.1 11.2	– 0.89 (0.71–1.10)
ATALANTE/ENGOT‐ov29 (NCT02891824)	PSROC (614)	1. Platinum‐based CX + Bev + Placebo + Maint (Bev + Placebo) 2. Platinum‐based CX + Bev + Atezolizumab + Maint (Bev + Atezolizumab)	III	11.3 13.5	– 0.83 (0.69–0.99)
MEDIOLA (NCT 02734004)	PSROC g*BRCA*m (51) Non‐g*BRCA*m (63)	1. Durvalumab + Olaparib (g*BRCA*m) 2. Durvalumab + Olaparib (non‐g*BRCA*m) 3. Bev + Durvalumab + Olaparib (non‐g*BRCA*m)	II	15.0 5.5 14.7	ORR 92.2 ORR 34.4 ORR 87.1
OPEB‐01/APGOT‐OV4 (NCT04361370)	PSROC Non‐*BRCA*m (44)	1. Maint (Bev + Pembrolizumab + Olaparib)	II	22.4	–
LEAP‐005 (NCT03797326)	PSROC, PRROC (31)	1. Lenvatinib + Pembrolizumab	II	6.2	ORR 26.0
Zsiros et al. (NCT02853318)	PSROC, PRROC (40)	1. Cyclophosphamide + Bev + Pembrolizumab	II	10.0	ORR 47.5
Liu et al. (NCT02853318)	PSROC, PRROC (38)	1. Bev + Nivolumab	II	8.1	ORR 28.9
OPAL Cohort A (NCT03574779)	PRROC (41)	1. Bev + Dostarlimab + Niraparib	II	7.9	ORR 17.1
NINJA (JapicCTI‐153 004)	PRROC (316)	1. Gemcitabine or Pegylated Liposomal Doxorubicin 2. Nivolumab	III	3.8 2.0	– 1.50 (1.20–1.90)
JAVELIN Ovarian 200 (NCT02580058)	PRROC (566)	1. Pegylated Liposomal Doxorubicin 2. Avelumab 3. Pegylated Liposomal Doxorubicin + Avelumab	III	3.5 1.9 3.7	– 1.68 (1.32–2.60) 0.78 (0.59–1.24)
Lee et al. (NCT02865811)	PRROC (26)	1. Pegylated Liposomal Doxorubicin + Pembrolizumab	II	5.6	ORR 26.1
TOPACIO/KEYNOTE‐162 (NCT02657889)	PRROC (62)	1. Pembrolizumab + Niraparib	I/II	3.4	ORR 18.0
MOCCA/APGOT‐OV2/GCGS‐OV3 (NCT03405454)	PSROC, PRROC OCCC (47)	1. Physician's choice CX 2. Durvalumab	II	3.5 1.9	ORR 18.8 ORR 10.7
PEACOCC (EudraCT 2017–004168‐36)	PSROC, PRROC OCCC (48)	1. Pembrolizumab	II	3.1	ORR 25.0

Abbreviations: Bev, bevacizumab; CX, chemotherapy; g*BRCA*m, germline *BRCA* mutation; HR, hazard ratio; ICI, immune checkpoint inhibitor; Maint, maintenance; mPFS, median progression‐free survival; OCCC, ovarian clear cell carcinoma; ORR, objective response rate; PRROC, platinum‐resistant recurrent ovarian cancer; PSROC, platinum‐sensitive recurrent ovarian cancer.

As investigated in the first‐line treatment setting, the triplet regimen (bevacizumab plus ICIs plus olaparib) was tested as either a treatment or maintenance therapy in the MEDIOLA or OPEB‐01/APGOT‐OV4 trials in PSROC without *BRCA* mutation.^98^, ^99^ Patients who received the triplet regimen (bevacizumab plus durvalumab plus olaparib) as the treatment setting in the MEDIOLA phase II study had a higher ORR than those who did not receive durvalumab (bevacizumab plus olaparib) (87% vs. 34%). Consistent with this, the phase II OPEB‐01/APGOT‐OV4 trial showed that the triplet regimen of bevacizumab plus pembrolizumab plus olaparib in the maintenance setting resulted in a promising outcome, with an mPFS of 22.4 months. In PRROC, the combination of bevacizumab, dostarlimab, and niraparib in cohort A of the OPAL phase II trial showed moderate activity with an ORR of 17.1%, with no association with HRD, *BRCA*, or PD‐L1 status.^100^

In the recurrent setting of ovarian cancer, different combinations of the anti‐angiogenic agents, including bevacizumab and lenvatinib with ICIs, have been tested.^101^, ^102^, ^103^ Among these, the combination therapy of bevacizumab, pembrolizumab, and oral metronomic cyclophosphamide showed good tolerability and was relatively effective, with an ORR of 47.5% in these patients. Phase III trials in the PRROC population with unmet clinical need include the JAVELIN Ovarian 200 trial in addition to the NINJA trial mentioned above.^104^ Results from the phase III JAVELIN Ovarian 200 trial showed that neither avelumab alone nor in combination with pegylated liposomal doxorubicin significantly improved PFS or OS compared to pegylated liposomal doxorubicin alone in the study population. However, the phase II trial testing the combination of pembrolizumab with pegylated liposomal doxorubicin in the same setting showed evidence of clinical benefit with an ORR of 26%, which was higher than the historical comparison of both.^105^ This aside, patients with PRROC who received pembrolizumab in combination with niraparib in the phase I/II TOPACIO/KEYNOTE‐162 trial showed an ORR of 18%, with no differences between *BRCA* mutation or HRD status.^106^

The optimal treatment of recurrent ovarian clear cell carcinoma (OCCC) remains a challenge. It has been suggested that patients with OCCC may benefit from treatment with ICIs.^107^ The randomized phase II MOCCA/APGOT‐OV2/GCGS‐OV3 trial comparing single‐agent chemotherapy with durvalumab in patients with recurrent OCCC did not show improved outcomes in the ICI arm.^108^ On the other hand, the PEACOCC study showed that ICI monotherapy with pembrolizumab in heavily pretreated patients with advanced OCCC showed encouraging responses with an ORR of 25%.^109^

Accordingly, the role of ICIs in the treatment of EOC remains limited. The immunosuppressive nature of the tumor microenvironment, the lack of predictive biomarkers, and tumor heterogeneity pose significant challenges. A deeper understanding of the immunobiology of EOC may pave the way for improved outcomes through the use of ICIs. Therefore, continued efforts to personalize immunotherapy based on patient‐specific factors will be essential for the future success of ICIs in the treatment of EOC.

## 5 ICIs IN RARE GYNECOLOGIC CANCERS

### 5.1 Vulvar and vaginal cancers

Vulvar and vaginal cancers account for 4% and 1%–2% of gynecological malignancies respectively,^110^, ^111^ and the 5‐year survival rate for locally advanced or metastatic disease is only 47.1% and 22.1% respectively (Table 9).^112^ As these cancers are rare and most patients are elderly, conducting large‐scale randomized controlled trials is challenging. Management is often extrapolated from cervical cancer, as both are primarily HPV‐related.

**TABLE 9:** Selected studies on ICIs in rare gynecologic cancers.

Trials	Cell types	Eligibility (*n*)	Arms	Phase	Primary endpoint	ORR (%)	DOR (months)	mPFS (months)	mOS (months)
Vulvar/vaginal cancer									
*Recurrence after first‐line therapy*									
Checkmate‐358 (NCT02488759)	Vulvar/vaginal SCC	5	1. Nivolumab	I/II	ORR	20	5.0	6‐month PFS rate: 40%	18‐month OS rate: 20%
KEYNOTE‐028 (NCT02054806)	Vulvar SCC PD‐L1 positive	18	1. Pembrolizumab	Ib	ORR	6		3.1	3.8
KEYNOTE‐158 (NCT02628067)	Vulvar SCC	101	1. Pembrolizumab	II	ORR	10.9	20.4	2.1	6.2
DART SWOG 1609 (NCT02834013)	Resistant/refractory vulvar cancer, (after ≥1 line)	16	1. Nivolumab + Ipilimumab	II, basket (cohort 47)	ORR	18.8	>1 year	2.2 12‐month PFS rate: 20%	7.6 12‐month OS rate: 44%
NCT0272173	Vulvar/vaginal SCC	3	1. Pembrolizumab	II	Non‐progression rate at 27 weeks	66.7			
NCT04430699	First line unresectable, locally advanced, or metastatic vulvar SCC	14; planned: 24	1. Cisplatin + Pembrolizumab + Radiation	II	ORR				
VolATIL (NCT03946358)	Advanced/metastatic vulvar cancer, HPV‐related (after ≥1 line)	Planned: NA (total 47 in all cohorts)	1. Atezolizumab + UCPVax vaccine	II	ORR at 4 months	Active, not recruiting			
APOLLO (NCT05761132)	Primary vSCC	Planned: 40	1. Pembrolizumab	II	ORR and intratumoral T‐cell population	Not yet recruiting			
NCT03452332	Recurrent/metastatic vulvar/vaginal cancer, HPV‐related (after ≥1 line)	Planned: NA (total 20 in all cohorts)	1. Tremelimumab + Durvalumab + SABR	I	Safety	Completed recruitment			
Cervical/vaginal/vulvar melanoma									
NCT05111574	Advanced recurrent cervical/vaginal/vulvar melanoma	Planned: 99	1. Nivolumab 2. Nivolumab + Cabozantinib	II	Recurrence‐free survival	Recruiting			
Gestational trophoblastic neoplasia									
*Recurrence after first‐line therapy*									
TROPHIMMUN (NCT03135769)	Resistant/refractory GTN (after ≥1 line)	Cohort A: 15	1. Avelumab	II	ORR (rate of hCG normalization)	46.7		NR	NR
		Cohort B: 7				14.3		1.4	50.7
DART SWOG 1609 (NCT02834013)	Resistant/refractory GTN (after ≥1 line)	4	1. Nivolumab + Ipilimumab	II, basket (cohort 47)	ORR	75	NR	NR 6‐month PFS: 75%	NR (no deaths)
CAP 01 (NCT0404701)	Resistant/refractory GTN (after ≥2 lines)	20	1. Camrelizumab + Apatinib	II	ORR	55	NR	9.5	NR 12‐month OS rate: 90%
TROPHAMET (NCT04396223)	Low‐risk GTN	22	1. Avelumab with methotrexate	I/II	ORR (rate of hCG normalization)	96.2			
CSEM018 (NCT04812002)	Resistant/refractory GTN (after ≥2 lines)	Planned: 20	1. Camrelizumab + Bevacizumab	II	PFS up to 2 years	Recruiting			
IMMUNORARE5 (NCT06790706)	Resistant/refractory GTN (after ≥1 line of combination chemotherapy)	Planned: NA (total 157 in all cohorts)	1. Domvanalimab + Zimberelimab	II, basket (cohort 2)	ORR (rate of hCG normalization) at 24 weeks	Not yet recruiting			
RESOLVE (NCT05635344)	Low‐risk post‐molar GTN before 2nd evacuation	Planned: 20	1. Pembrolizumab 2. None	II, basket (cohort 2)	Proportion of patients consented to study and who complete treatment	Recruiting			

Abbreviations: Act‐D, actinomycin D; DOR, duration of response; iv, intravenous; mOS, median overall survival; mPFS, median progression‐free survival; MTX, methotrexate; NA, not applicable; NR, not reached; ORR, objective response rate; OS, overall survival; SABR, stereotactic ablative radiotherapy; SCC, squamous cell carcinoma.

Similar to cervical cancer, CheckMate‐358 was the first study using ICIs in HPV‐related vulvar and vaginal cancer and demonstrated an ORR of 20%.^32^ The KEYNOTE‐158 trial was the largest study, enrolling 101 patients with vulvar SCC.^113^ The ORR was 10.9% (95% CI 5.6–18.7) among all patients. Patients with PD‐L1 expression had a lower ORR of 9.5% (95% CI 4.2–17.9) compared to those without PD‐L1 expression (ORR 28.6%, 95% CI 3.7–71.0), though the latter group consisted of seven patients only. The pooled results of KEYNOTE‐158 and KEYNOTE‐028 showed that the pooled ORR was 10% (95% CI 0.00–0.84) and 9% (95% CI 0.00–89), respectively, in patients with PD‐L1 expression.^114^ Even in the ITT population, the 12‐month PFS and OS rate was 9% (95% CI 0–85) and 33% (95% CI 4–85), respectively. On the other hand, studies have shown that HPV‐independent SCC, typically arising from differentiated vulvar intraepithelial neoplasia, exhibits higher expression than HPV‐dependent SCC.^115^ However, the KEYNOTE‐158 and KEYNOTE‐028 trials did not clarify the relationship between HPV status and PD‐L1 expression. Therefore, further translational research is required to identify the candidates who may benefit most from ICI treatment.

### 5.2 Cervical/vaginal/vulvar melanoma

Patients with genital tract melanoma (GTM) have a poor prognosis.^116^, ^117^ Their PD‐L1 expression is in the range of 0%–56%,^117^, ^118^, ^119^ and up to 46% of patients with vulvovaginal melanoma had high stromal tumor‐infiltrating lymphocytes.^120^ PD‐L1 expression and median CD8 tumor‐infiltrating lymphocyte density appeared to be higher in vaginal and vulvar melanoma compared to cervical melanoma.^117^ Most studies using ICIs were limited to case reports or case series. There were multiple reports showing the clinical efficacy of ICIs in GTM,^121^ including one pooled analysis demonstrating an ORR up to 37.1% (95% CI 21.5–55.1) in mucosal melanoma including GTM with the use of nivolumab and ipilimumab.^122^ However, one case series compared 75 patients using ICIs and another 225 patients without ICIs, demonstrating that the use of ICIs did not prolong OS (HR 0.81, 95% CI 0.57–1.14).^123^ Due to the rarity of the disease, there is only one ongoing phase II trial investigating the use of nivolumab with or without cabozantinib in GTM (NCT05111574).

### 5.3 Gestational trophoblastic neoplasia

Although high‐risk gestational trophoblastic neoplasia (GTN) can be treated using combination chemotherapy with survival rates up to 80%–90%,^124^ there are few treatment options for resistant/refractory diseases. PD‐L1 is strongly expressed in syncytiotrophoblasts in normal placenta, molar pregnancy, and choriocarcinoma, and with variable but weaker expression in intermediate trophoblastic neoplasms, placental site trophoblastic tumors, and epithelioid trophoblastic tumors,^125^, ^126^, ^127^, ^128^ and 22C3 appeared to an appropriate antibody.^129^ PD‐1‐positive lymphocytes were also identified in the implantation site and trophoblastic tumor.^126^ This provided the rationale for using ICIs in GTN.

A retrospective study of 66 patients showed that the ORR was 62.9% using anti‐PD1 alone, and 96.8% when combined with chemotherapy.^130^ There were several phase II trials on using immunotherapy in recurrent/resistant GTN. The ORR was 47% with avelumab alone,^131^ 55% when using camrelizumab and apatinib,^132^ and 75% when using nivolumab and ipilimumab.^133^ However, most of the responders were limited to recurrent post‐molar and choriocarcinoma, and the response in placental site trophoblastic tumors and epithelioid trophoblastic tumors remained poor. In addition, due to the rarity of this disease entity, these studies were limited to phase II trials with a small sample size. There are a few ongoing phase II trials, including one using combined avelumab and methotrexate in low‐risk GTN.^134^

## 6 CONCLUSION

The role of ICIs in gynecologic cancers is evolving, with multiple ongoing clinical trials investigating the use of different drug combinations and optimal dosage strategies. Tumors with low immunogenicity, such as ovarian cancer, frequently demonstrate resistance to ICIs. Consequently, combinations with angiogenesis inhibitors, PARPi, chemotherapy, or ADC are required to enhance response rates. Furthermore, key biomarkers, such as PD‐L1 expression, TMB, and MSI status, play a pivotal role in identifying patients who could benefit most from this treatment. However, these biomarkers still have drawbacks. In addition, irAEs can be a significant concern, particularly in patients with pre‐existing autoimmune conditions, necessitating meticulous management and monitoring throughout ICI therapy. There is an urgent need to explore novel combination therapies, particularly for cold tumors, as well as to identify more reliable biomarkers for predicting patient response and develop strategies for anticipating and managing various irAEs.

## AUTHOR CONTRIBUTIONS

All authors contributed to the development of the current review and approved the final version.

## FUNDING INFORMATION

This research received no external funding.

## CONFLICT OF INTEREST STATEMENT

The authors have no conflicts of interest.

## Data Availability

Data sharing is not applicable to this article as no new data were created or analyzed in this study.
